# Physics-Informed Side-Scan Sonar Perception: Tackling Weak Targets and Sparse Debris via Geometric and Frequency Decoupling

**DOI:** 10.3390/s26061938

**Published:** 2026-03-19

**Authors:** Bojian Yu, Rongsheng Lin, Hanxiang Zhou, Jianxiong Zhang, Xinwei Zhang

**Affiliations:** 1 Jurong Petroleum and Natural Gas University Enterprise Joint Laboratory, Zhejiang Ocean University, Zhoushan 316022, China; 20252028@zjou.edu.cn (B.Y.);; 2 School of Marine Engineering Equipment, Zhejiang Ocean University, Zhoushan 316022, China; 3 Jurong Energy (Xinjiang) Co., Ltd., Bayingolin Mongol Autonomous Prefecture 841600, China; 4 School of Foreign Languages, Zhejiang Ocean University, Zhoushan 316022, China

**Keywords:** autonomous underwater search and rescue, side-scan sonar, acoustic shadow, wavelet transform, graph reasoning, physics-guided learning

## Abstract

Side-scan sonar (SSS) serves as the primary perceptual instrument for Autonomous Underwater Vehicles (AUVs) in large-scale marine search and rescue (SAR) operations. However, the detection of critical targets is frequently hindered by severe hydro-acoustic noise, the spatial discontinuity of wreckage, and the weak visual signatures of small targets. To surmount these challenges, this paper presents WPG-DetNet. First, we introduce a Wavelet-Embedded Residual Backbone (WERB) to reconstruct the conventional downsampling paradigm. By substituting standard pooling with the Discrete Wavelet Transform (DWT), this architecture explicitly disentangles high-frequency noise from structural information in the frequency domain, thereby achieving the adaptive preservation of edge fidelity for large human-made targets while filtering out speckle interference. Then, addressing the distinct challenge of discontinuous aircraft wreckage, the framework further incorporates a Debris Graph Reasoning Module (D-GRM). This module models scattered fragments as nodes in a topological graph to capture long-range semantic dependencies, transforming isolated instance recognition into context-aware scene understanding. Finally, to bridge the gap between AI and underwater physics, we design a Shadow-Aided Decoupling Head (SADH) equipped with a physics-informed geometric loss. By enforcing mathematical consistency between target height and acoustic shadow length, this mechanism establishes a rigorous discriminative criterion capable of distinguishing weak-echo human bodies from seabed rocks based on shadow geometry. Experiments on the SCTD dataset demonstrate that WPG-DetNet achieves a mean Average Precision ($mAP_{50}$) of 97.5% and a Recall of 96.9%. Quantitative analysis reveals that our framework outperforms the classic Faster R-CNN by a margin of 12.8% in $mAP_{50}$ and surpasses the Transformer-based RT-DETR-R18 by 5.6% in high-precision localization metrics ($mAP_{50:95}$). Simultaneously, WPG-DetNet maintains superior efficiency with an inference speed of 62.5 FPS and a lightweight parameter count of 16.8 M, striking an optimal balance between robust perception and the real-time constraints of AUV operations.

## 1. Introduction

Side-scan sonar (SSS) stands as a pivotal technology in marine observation, transforming acoustic backscatter into high-resolution seafloor imagery [1]. This capability has not only significantly expanded the frontiers of our understanding of the deep-sea environment but also serves as the technological backbone for diverse marine operations [2]. SSS plays an indispensable role across diverse marine operations. Its applications range from bathymetric mapping and resource exploration to shipwreck salvage and subsea engineering safety across both scientific research and practical engineering applications [3]. However, the automation of object detection in SSS imagery remains fraught with challenges, constrained by the operational limitations of acquisition equipment and the inherent complexity of the marine environment [4]. Unlike terrestrial optical photography, SSS imagery possesses unique acoustic characteristics governed by distinct imaging mechanisms [5]. First, the complexity of the underwater acoustic channel results in low resolution and poor contrast. In this study, speckle noise refers to the granular noise inherent in coherent acoustic imaging, while reverberation denotes the interference from seabed scattering, which obscures textural details and blur object boundaries. Second, intensity inhomogeneity arises from the sonar’s reliance on echo intensity, causing the visual signature of targets to vary drastically with incidence angle and seafloor substrate, thereby inducing significant intra-class variability. Furthermore, targets in underwater scenarios—such as mines or human bodies—often manifest as “weak objects” with diminutive dimensions and faint features, easily submerged within complex reverberation noise, reef backgrounds, or high-contrast acoustic shadows. Consequently, standard detection models designed for natural optical images often flounder when directly applied to this acoustic domain.

Nevertheless, the rapid evolution of deep learning has catalyzed the adoption of such algorithms in underwater sonar image processing [6]. Early paradigms predominantly leveraged Convolutional Neural Networks (CNNs), such as the YOLO series and Mask R-CNN. Although these methods exploit the robust feature extraction of CNNs, they typically transplant architectures designed for natural images, thereby neglecting the specific frequency domain attributes of sonar data designed for natural images, often neglecting specific attributes of sonar data, such as frequency domain characteristics [7]. Moreover, the inherent local receptive field of CNNs limits their ability to capture global context, leading to missed detections or false positives when processing minute targets against complex backgrounds [8]. Recently, the emergence of Vision Transformers—exemplified by end-to-end detectors like DETR—has drawn significant attention for their ability to model global dependencies via self-attention mechanisms, while Transformers offer superior advantages in handling long-range dependencies compared to CNNs, their direct application to sonar imagery faces distinct limitations: on the one hand, the high computational cost and slow convergence rates impede real-time underwater deployment; on the other hand, existing Transformer architectures struggle with the feature extraction and interaction required for the blurred, indistinct targets typical of sonar imagery, thereby bottlenecking further performance gains.

Based on the aforementioned issues, this paper proposes the WPG-DetNet model. The main research and innovations of this paper can be summarized as follows:

(1) Wavelet Domain-Based Feature Disentanglement Strategy: We propose a Wavelet-Embedded Residual Backbone that redefines the downsampling paradigm for sonar images. By replacing conventional pooling operations with the Discrete Wavelet Transform (DWT), the architecture explicitly decouples high-frequency speckle noise from low-frequency structural information in the frequency domain. This innovation effectively suppresses speckle noise while maximally preserving edge fidelity for large artificial targets, such as shipwrecks.
(2) Topological Reasoning Mechanism for Discrete Targets: We introduce a Debris Graph Reasoning Module (D-GRM) to address the challenge of spatially discontinuous distributions of aircraft wreckage fragments. By modeling potential candidate objects as nodes in a topological graph, the module employs Graph Convolutional Networks (GCNs) to capture long-range spatial dependencies and semantic correlations. This approach transforms the detection task from isolated instance identification into context-aware scene understanding, significantly reducing the missed detection rate for discrete fragments.
(3) Physics-Informed Geometric Verification Method: We design a Shadow-Aided Decoupling Head to bridge the gap between deep learning and underwater acoustic physics. This module incorporates a Physics-Informed Geometric Loss, which enforces mathematical consistency between target height and acoustic shadow length. This consistency serves as a critical criterion to differentiate weak-echo human targets from complex seabed rocks based on shadow characteristics.

The remainder of this manuscript is organized as follows: Section 2 reviews the prevailing literature on deep learning-based object detection and side-scan sonar image analysis. Section 3 delineates the methodological framework of WPG-DetNet, detailing the Wavelet-Embedded Backbone, Debris Graph Reasoning Module, and Shadow-Aided Decoupling Head. Section 4 presents a comprehensive empirical analysis, substantiating the model’s performance through comparative and ablation studies. Section 5 discusses the interpretability and robustness of the proposed framework. Finally, Section 6 concludes the study and outlines potential avenues for future research.

## 2. Related Work

### 2.1. Sonar Image Analysis

SSS serves as a critical instrument for underwater environmental perception and large-scale seabed surveys [9]. Its core value lies in its ability to provide high-resolution two-dimensional acoustic imagery, enabling the fine-grained characterization of seabed topography and substrate types [10]. In practical applications, SSS plays a pivotal role in underwater emergency Search and Rescue (SAR). It has become the premier method for confirming crash sites and debris distribution, particularly in the search and localization of crashed aircraft wreckage, owing to its wide coverage and high-definition imaging capabilities [11].

**Figure 1:**
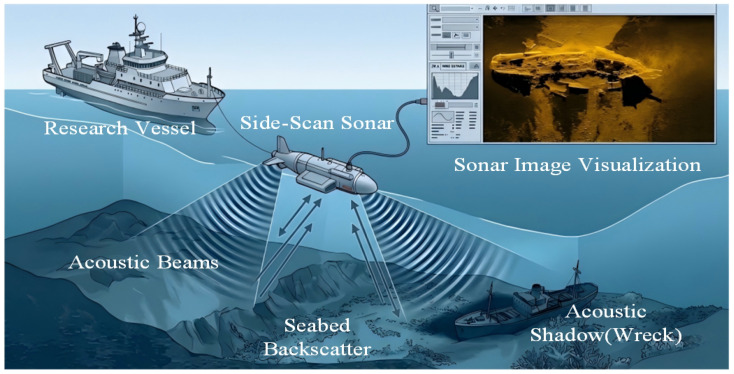
Illustration of the SSS data acquisition process and acoustic shadow formation.

As illustrated in Figure 1, the SSS detection system primarily consists of a surface vessel and a submerged towfish. During operation, the vessel tows the towfish, equipped with transducer arrays, through the near-bottom water column via an armored cable. The transducers periodically emit high-frequency fan-shaped acoustic beams perpendicular to the track. These beams feature a wide vertical opening angle to cover a broad swath range, while maintaining an extremely narrow horizontal beamwidth to ensure high along-track spatial resolution.

Upon striking the seabed, the acoustic pulses generate backscatter signals of varying intensities depending on the acoustic impedance and microscopic roughness of the medium. High-impedance artificial hard targets, such as shipwrecks, strongly reflect acoustic waves, appearing as highlighted areas on the sonar image. Simultaneously, due to the rectilinear propagation of sound waves, objects protruding from the seabed obstruct the acoustic path, creating a zone with no return signal behind the target. This results in a distinct black acoustic shadow on the image. This “highlight-shadow” pairing not only clearly delineates the geometric profile and spatial distribution of the shipwreck but also allows for the estimation of the target’s approximate height based on the geometric imaging relationship (triangulation of slant range, towfish altitude, and shadow length), thereby achieving high-precision detection and identification of shipwrecks and micro-topographic features in complex underwater environments.

### 2.2. Instance Detection of Natural Images and Sonar Images

The advent of artificial intelligence (AI) is catalyzing a paradigm shift in our understanding and exploitation of the ocean, advancing the field with unprecedented depth and breadth [12]. Currently, the integration of AI into diverse frontiers—ranging from remote sensing interpretation and acoustic signal processing to numerical model parameterization and the control of autonomous platforms—is yielding transformative breakthroughs [13,14]. For instance: Sethuraman et al. [15] addressed the critical challenge of data scarcity in underwater robotics by introducing the AI4Shipwrecks dataset, which provides a high-resolution benchmark for shipwreck segmentation to advance autonomous sonar image understanding. To ensure data reliability, Tolie et al. [16] developed a blind image quality assessment method that leverages micro- and macro-scale wavelet features to quantify degradation in sonar imagery, effectively mapping perceptual quality to objective scores. In the realm of acoustic surveillance, da Silva et al. [17] evaluated supervised learning algorithms for passive sonar signal analysis, demonstrating that models like XGBoost offer the robust accuracy and computational efficiency required for real-time ship classification. Furthermore, Orescanin et al. [18] enhanced the robustness of Synthetic Aperture Sonar (SAS) perception by applying Bayesian deep learning to classify imaging artifacts, utilizing uncertainty quantification to improve the reliability of autonomous underwater systems.

Although technologies such as remote sensing and acoustic monitoring have generated a deluge of multi-modal marine data, progress remains constrained by the “feature engineering bottleneck.” Conventional machine learning relies heavily on hand-crafted features (e.g., texture and shape), which are defined by domain experts. This process is not only labor-intensive and subjective but also ill-equipped to capture the complex, non-linear relationships inherent in the data.

In recent years, the evolution of deep learning has catalyzed a fundamental paradigm shift from hand-crafted feature engineering to end-to-end feature learning. These models possess the unique capability to automatically extract discriminative, hierarchical representations directly from raw data, such as pixel matrices and time series.

Specifically, the evolution of general object detection in deep learning is primarily characterized by two dominant paradigms: two-stage and one-stage detectors. Two-stage frameworks, epitomized by the R-CNN series (e.g., Faster R-CNN, Mask R-CNN), operate by first generating region proposals, followed by a refinement phase for classification and bounding box regression. For example, Xu et al. [19] proposed the Active Mask-Box Scoring R-CNN to address the mismatch between positioning accuracy and classification confidence often found in sonar instance segmentation. By incorporating a boxIoU head to predict bounding box quality and employing a Triplets-measure-based Active Learning (TBAL) framework, they effectively tackled the high annotation costs inherent to sonar imagery. Similarly, to enhance the perception capabilities of Unmanned Underwater Vehicles (UUV), Xiao et al. [20] developed the SDA-Mask R-CNN framework. This architecture integrates a Structural Synergistic Group-Attention Residual Network (SSGAR-Net) and a Depth-Weighted Hierarchical Fusion Network (DWHF-Net) to optimize information flow and multi-scale feature fusion, successfully mitigating challenges related to low segmentation accuracy and ambiguous edge delineation in seabed terrain analysis.

While these architectures offer benchmark-level accuracy and localization, their multi-stage nature entails high computational costs.

In contrast, single-stage detectors like YOLO, SSD, and RetinaNet treat object detection as a single-shot regression problem. By eliminating the proposal generation step, these models achieve real-time inference, satisfying the demands of latency-sensitive applications. For example, Li et al. [21] focused on real-time AUV perception by proposing the MA-YOLOv7 network, which integrates multi-scale information fusion and attention mechanisms with threshold segmentation to achieve high recall and rapid processing speeds. Addressing limited computational resources, the MAL-YOLO study introduced a lightweight architecture combining depthwise separable convolutions with an Efficient Multi-scale Attention (EMA) module, significantly reducing model complexity while maintaining high detection accuracy. Exploring advanced generative integration, Wen et al. [22] combined diffusion models with an improved YOLOv7 architecture, utilizing the strong feature extraction capabilities of diffusion processes to enhance target detection robustness in complex environments. Focusing on model optimization, Aubard et al. [23] investigated knowledge distillation techniques within the YOLOX-ViT framework, aiming to improve the efficiency and performance of sonar object detectors through advanced teacher-student learning strategies. Finally, tackling the fundamental bottleneck of data scarcity, Li et al. [24] developed novel image generation algorithms like UA-CycleGAN and ADA-StyleGAN3, creating high-quality synthetic samples to effectively train these deep learning models under zero- and few-sample conditions.

Despite the proven success of these architectures in the realm of optical imaging, their direct transfer to the underwater domain often yields suboptimal performance. This is primarily attributed to the significant domain gap between in-air optical scenes and the complex underwater hydroacoustic environment.

### 2.3. Graph Neural Networks and Physics-Informed Learning

While standard CNNs excel at extracting local visual features, they fundamentally lack the capacity to model long-range topological dependencies and adhere to physical constraints—limitations that are particularly acute in the underwater domain. GCNs have revolutionized the modeling of non-Euclidean data by treating objects as nodes within a topological space rather than pixels on a rigid grid. In terrestrial computer vision, this paradigm is pivotal for capturing long-range semantic dependencies (e.g., scene graph generation); however, the potential of topological reasoning remains largely underexplored in underwater contexts.

Current SSS detectors rely heavily on CNNs that process local textures but fail to contextualize scattered targets. For instance, debris from airplane wreckage forms an irregular geometry that is semantically connected yet spatially dispersed. Existing methods treat these fragments as isolated instances, often failing to aggregate such context, which leads to missed detections in complex debris fields.

Concurrently, physics-informed learning has emerged as a robust strategy to enhance model generalizability by explicitly embedding physical laws. In SSS imagery, acoustic shadow formation is governed by strict geometric principles: shadow length is a deterministic function of target height, sensor altitude, and slant range. Despite these rigorous relationships, standard data-driven approaches often neglect these priors, treating shadows merely as visual textures (i.e., dark regions) rather than quantitative geometric evidence. This oversight renders detectors vulnerable to false alarms caused by seabed protrusions like rocks. By incorporating physics-based geometric constraints, we aim to bridge this gap, leveraging shadow consistency as a decisive discriminator to verify weak-echo targets.

Ultimately, by integrating topological reasoning with acoustic physics priors, our framework addresses these systemic deficiencies in current underwater perception.

## 3. Methods

### 3.1. Overall Architecture

**Figure 2:**
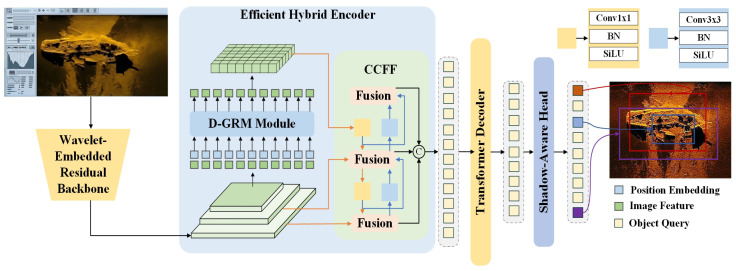
WPG-DetNet overall architecture.

To address challenges such as severe speckle noise interference and insufficient utilization of acoustic shadows in side-scan sonar images, this paper proposes an improved object detection model named WPG-DetNet, based on RE-DETR. As illustrated in Figure 2, the architecture diagram shows that the model primarily consists of three core components: feature extraction, encoding enhancement, and a domain-specific detection head.

First, in the feature extraction stage, the model employs a Wavelet-Embedded Residual Backbone. Unlike conventional CNN backbones, this module integrates wavelet transform to extract salient target features from raw sonar images. This design effectively suppresses prevalent speckle noise in underwater sonar imagery while preserving critical high-frequency information such as target edges and textures in the frequency domain.

Second, to enhance feature representation, the model incorporates an Efficient Hybrid Encoder. This encoder integrates a D-GRM Module and a CCFF structure. Specifically, the D-GRM module captures global long-range dependencies, while the CCFF structure facilitates the Concatenation and interaction of multi-scale features through hierarchical fusion units. This hybrid design not only strengthens the model’s perception of multi-scale targets but also retains global contextual information, effectively mitigating the loss of local detailed features during network propagation.

Finally, in the detection and localization stage, the model features a novel Shadow-Aware Head appended to the Transformer decoder. Leveraging the physical “target-shadow” duality inherent to sonar imaging, this detection head focuses not only on target echo characteristics but also utilizes acoustic shadow features for auxiliary localization. This advancement addresses the oversight of shadows in conventional detection heads, thereby enhancing localization precision and classification accuracy for targets such as shipwrecks in complex seabed environments.

### 3.2. Wavelet-Embedded Residual Backbone

In underwater SSS imaging, object detection faces two primary challenges: severe speckle noise that obscures edge details, and the irreversible loss of fine textures caused by traditional downsampling operations (e.g., max pooling) in CNNs.

**Figure 3:**
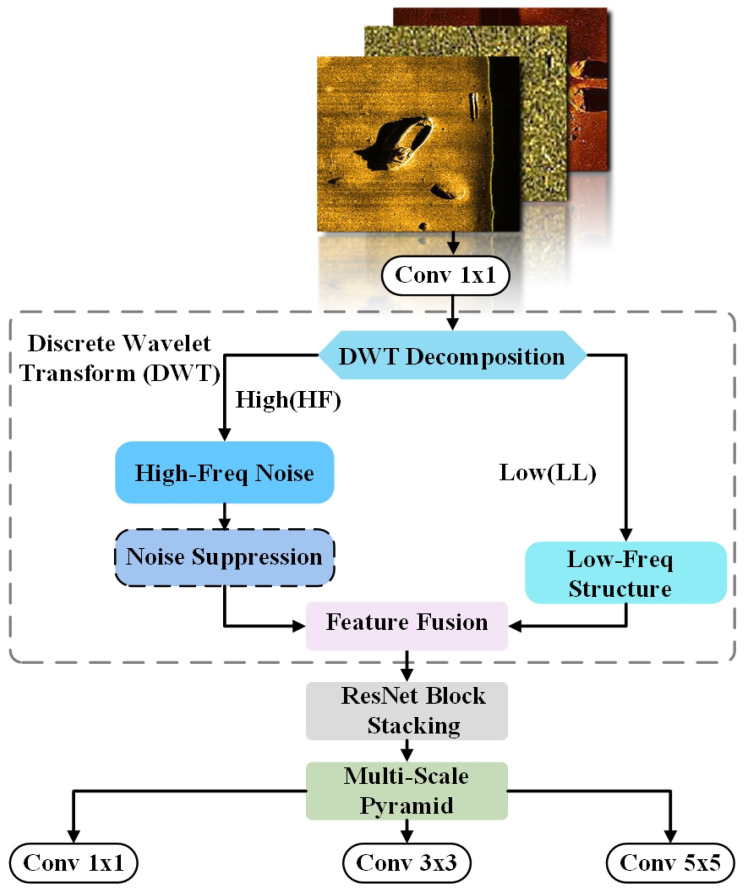
Wavelet-Embedded Residual Backbone.

To address these limitations, we propose the Wavelet-Embedded Residual Backbone (WERB). As illustrated in Figure 3. The core innovation lies in utilizing the DWT to replace standard spatial downsampling, thereby extending feature extraction from the spatial domain to the time-frequency domain. By explicitly decoupling the signal into low-frequency structural components and high-frequency detail components, WERB effectively suppresses noise while preserving critical structural information such as shipwreck contours.

The Haar wavelet is employed for its minimal computational overhead and its ability to preserve sharp transitions, which is crucial for maintaining the structural boundaries of human-made underwater targets. Compared to higher-order continuous wavelets like Daubechies, which tend to smooth out abrupt changes, the Haar basis is more effective at retaining the sharp acoustic edges of wreckage. Furthermore, a single-level decomposition is utilized to strike an optimal balance between expanding the receptive field and preventing the irreversible loss of fine textures inherent to extremely small targets.

Given an input feature tensor X$\in R^{C\times H\times W}$, where *C*, *H*, and *W* denote the channel number, height, and width, respectively. We introduce the 2D-DWT as a lossless downsampling operator. Using the Haar wavelet basis, the input X is decomposed into four complementary sub-bands:

(1) $$X_{LL},X_{LH},X_{HL},X_{HH}=\mathrm{DWT}\left(X\right)$$

where $X_{LL}\in R^{C\times \frac{H}{2}\times \frac{W}{2}}$ is the *Low-Frequency Approximation*, capturing the global topology and intensity distribution. The set $\left\{X_{LH},X_{HL},X_{HH}\right\}$ represents the *High-Frequency Details* corresponding to horizontal, vertical, and diagonal edges, respectively. In sonar imagery, speckle noise is predominantly concentrated within these high-frequency sub-bands.

To eliminate noise while retaining edges, we design a lightweight *Noise Suppression Subnet* targeting only the high-frequency components. The three high-frequency sub-bands are concatenated along the channel dimension to form $X_{HF}\in R^{3C\times \frac{H}{2}\times \frac{W}{2}}$, which is then processed by a suppression function $F_{suppress}$:

(2) $${\hat{X}}_{HF}=F_{suppress}\left(X_{HF}\right)=\sigma \left(\mathrm{BN}\left(W_{1\times 1}\left(\delta \left(\mathrm{BN}\left(W_{3\times 3}\left(X_{HF}\right)\right)\right)\right)\right)\right)$$

where W denotes convolution, BN is Batch Normalization, and δ and σ are ReLU and Sigmoid activation functions, respectively. The refined high-frequency features ${\hat{X}}_{HF}$ are then inversely fused with the unprocessed low-frequency features $X_{LL}$:

(3) $$X_{fusion}=W_{fusion}\left(\left[X_{LL},{\hat{X}}_{HF}\right]\right)$$

This step maps the feature dimension to 4C, reconstructing a complete time-frequency feature space. Subsequently, the fused features are fed into stacked ResNet blocks for deep semantic extraction.

To address the extreme scale variation in SSS targets (e.g., large shipwrecks vs. small human bodies), we integrate a parallel multi-scale pyramid structure at the end of the module. The output features Y are aggregated via kernels of varying receptive fields:

(4) $$\mathrm{Output}=\{\phi_{1\times 1}\left(Y\right),\phi_{3\times 3}\left(Y\right),\phi_{5\times 5}\left(Y\right)\}$$

This design ensures the network simultaneously perceives local details and global context.

To elucidate the data flow within the WERB module, we present the evolution of feature dimensions in Table 1, and the algorithmic process is detailed in Algorithm 1. This design allows WERB to perform 2× downsampling while utilizing 4× channel capacity to carry lossless spatial-frequency information, avoiding the information loss typical of pooling layers.
**Algorithm 1** Forward Process of Wavelet-Embedded Residual Backbone.**Require:** Input Tensor X with shape (B,C,H,W)**Ensure:** Multi-scale Features $\left[O_{1},O_{3},O_{5}\right]$1:$X\leftarrow {\mathrm{Conv}}_{1\times 1}\left(X\right)$▹ Pre-projection2:**Step 1: Frequency Decoupling (DWT)**3:$X_{LL},\left(X_{LH},X_{HL},X_{HH}\right)\leftarrow \mathrm{DWT}\left(X\right)$4:▹ Output shapes: (B,C,H/2,W/2)5:**Step 2: Noise Suppression**6:$X_{HF}\leftarrow \mathrm{Concat}\left(\left[X_{LH},X_{HL},X_{HH}\right],\dim=1\right)$7:$X_{HF}^{clean}\leftarrow \mathrm{NoiseSuppressionBlock}\left(X_{HF}\right)$8:▹ Operates on High-Freq channels only9:**Step 3: Feature Fusion**10:$X_{combined}\leftarrow \mathrm{Concat}\left(\left[X_{LL},X_{HF}^{clean}\right],\dim=1\right)$11:$X_{fused}\leftarrow \mathrm{ConvFusion}\left(X_{combined}\right)$12:▹ Channel mapping: $4C\to C^{\prime }$13:**Step 4: Deep Extraction**14:$X_{deep}\leftarrow \mathrm{ResNetStack}\left(X_{fused}\right)$15:**Step 5: Multi-Scale Pyramid**16:$O_{1}\leftarrow {\mathrm{Conv}}_{1\times 1}\left(X_{deep}\right)$17:$O_{3}\leftarrow {\mathrm{Conv}}_{3\times 3}\left(X_{deep}\right)$18:$O_{5}\leftarrow {\mathrm{Conv}}_{5\times 5}\left(X_{deep}\right)$19:**return**
 $\left[O_{1},O_{3},O_{5}\right]$

### 3.3. Debris Graph Reasoning Module

The distribution of aircraft wreckage on the seabed is characterized by ’spatial discontinuity but semantic consistency’, where conventional CNNs often struggle due to their limited local receptive fields.

**Figure 4:**
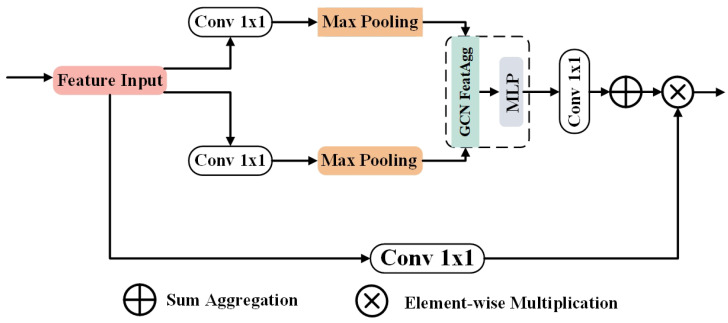
Debris Graph Reasoning Module.

To address this, we introduce the Debris Graph Reasoning Module (D-GRM). As illustrated in Figure 4, which projects feature maps from the coordinate space to a latent graph space, employs Graph Convolutional Networks (GCNs) to capture global topological dependencies, and re-projects the reasoned context back to the spatial domain.

Formally, let the input feature tensor be denoted as $X\in R^{C\times H\times W}$. The D-GRM processes *X* through a dual-branch structure via three distinct stages: projection, reasoning, and fusion. First, to transform the dense pixel grid into a sparse graph structure, we employ a max-pooling operation followed by a spatial flattening transformation. Let $F_{proj}\left(\cdot \right)$ denote the projection function; the node feature matrix $V\in R^{N\times C^{\prime }}$ is obtained as

(5) $$V=\mathrm{Flatten}(\mathrm{MaxPool}\left({\mathrm{Conv}}_{1\times 1}\left(X\right)\right))$$

where $N=H^{\prime }\times W^{\prime }$ represents the number of nodes, explicitly decoupling the feature representation from the rigid spatial grid.

Second, the extracted nodes are processed by the GCN unit to capture long-range dependencies. Let *A* be the adjacency matrix and $W_{g}$ be the learnable weights; the aggregated node features $V_{agg}$ are computed via $V_{agg}=\sigma \left({\tilde{D}}^{-\frac{1}{2}}\tilde{A}{\tilde{D}}^{-\frac{1}{2}}VW_{g}\right)$, followed by an MLP for channel-wise interaction to yield $V_{out}$.

Finally, to fuse the global context back into local features, we perform a reverse projection where the graph features are reshaped from the node domain (*N*) back to the spatial domain ($H^{\prime }\times W^{\prime }$). The global context attention map $M_{context}$ and the final output *Y* are defined as follows:

(6) $$M_{context}=\mathrm{Upsample}\left({\mathrm{Reshape}}_{N\to H^{\prime }\times W^{\prime }}\left({\mathrm{Conv}}_{1\times 1}\left(V_{out}\right)\right)\right)$$

(7) $$Y=\phi \left(X\right)⊗M_{context}$$

This mechanism ensures that the model selectively highlights discrete debris candidates by leveraging the reasoned global topology. The algorithmic process is detailed in Algorithm 2, and the tensor evolution is listed in Table 2.
**Algorithm 2** Processing Logic of Debris Graph Reasoning Module.**Require:** Input Feature Tensor $X\in R^{B\times C\times H\times W}$**Ensure:** Enhanced Feature Tensor *Y*1:**Step 1: Node Extraction**2:$F_{proj}\leftarrow {\mathrm{Conv}}_{1\times 1}\left(X\right)$▹ Channel reduction: $C\to C^{\prime }$3:$V_{grid}\leftarrow \mathrm{MaxPooling}\left(F_{proj}\right)$▹ Downsample spatial dims4:$V_{nodes}\leftarrow \mathrm{Flatten}\left(V_{grid}\right)$▹**Reshape**: $\left(B,C^{\prime },H^{\prime },W^{\prime }\right)\to \left(B,C^{\prime },N\right)$5:**Step 2: Topological Reasoning**6:$V_{agg}\leftarrow \mathrm{GCN}\_\mathrm{FeatAgg}\left(V_{nodes}\right)$▹ Global info propagation7:$V_{out}\leftarrow \mathrm{MLP}\left(V_{agg}\right)$▹ Channel mixing8:**Step 3: Attention Map Generation**9:$M_{flat}\leftarrow \mathrm{SumAgg}\left({\mathrm{Conv}}_{1\times 1}\left(V_{out}\right)\right)$10:$M_{spatial}\leftarrow \mathrm{Reshape}\left(M_{flat}\right)$▹**Restore grid**: $\left(B,C,N\right)\to \left(B,C,H^{\prime },W^{\prime }\right)$11:$A_{map}\leftarrow \mathrm{Upsample}\left(M_{spatial}\right)$▹ Align with input spatial resolution12:**Step 4: Context Fusion**13:$X_{trans}\leftarrow {\mathrm{Conv}}_{1\times 1}\left(X\right)$▹ Bottom branch transform14:$Y\leftarrow X_{trans}⊗A_{map}$▹ Element-wise weighting15:**return**
 *Y*

### 3.4. Shadow-Aided Decoupling Head

In the underwater acoustic domain, small-scale targets (e.g., human bodies) often exhibit weak echo intensities that are easily submerged in seabed reverberation, rendering standard appearance-based detectors ineffective.

**Figure 5:**
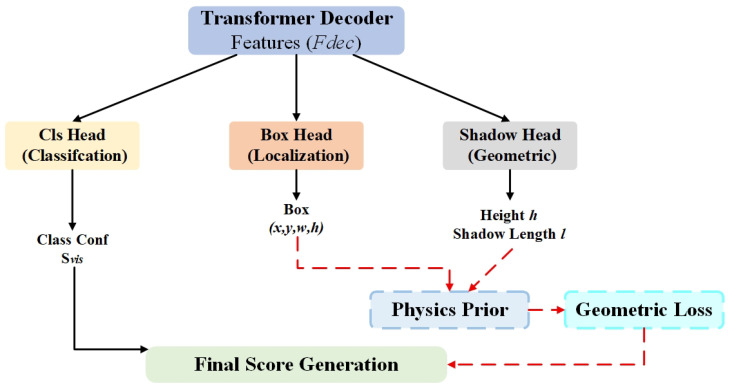
Architecture of the Shadow-Aided Decoupling Head (SADH).

To overcome this, we propose the Shadow-Aided Decoupling Head (SADH), as illustrated in Figure 5. This module bridges the gap between deep learning and underwater acoustic physics. Unlike conventional detection heads that rely solely on visual texture, SADH leverages the “highlight-shadow” duality inherent in sonar imaging, treating the acoustic shadow as rigorous geometric evidence rather than merely a background feature.

Formally, the formation of an acoustic shadow is governed by the triangulation of the sonar towfish altitude $H_{s}$, the target height $H_{t}$, and the slant range $R_{s}$. According to the geometric imaging principle, objects located further from the sonar (larger slant range) cast longer shadows. The theoretical shadow length $L_{theo}$ is proportional to the slant range and can be approximated as follows:

(8) $$L_{theo}\approx \frac{H_{t}\times R_{s}}{H_{s}}\;\left(\mathrm{assuming}\;H_{t}\ll H_{s}\right)$$

where $R_{s}$ denotes the slant range distance from the transducer to the target. Based on this prior, the SADH incorporates a parallel regression branch alongside the standard classification and bounding box heads. Let the input feature from the transformer decoder be $F_{dec}\in R^{B\times N_{q}\times C}$, where $N_{q}$ is the number of object queries. The module predicts the target height $\hat{h}$ and shadow length $\hat{l}$ simultaneously.

To enforce physical consistency, we introduce a Physics-Informed Geometric Loss ($L_{geo}$):

(9) $$L_{geo}={∥\hat{l}-\frac{\hat{h}\times R_{s}}{H_{s}}∥}_{2}^{2}$$

By minimizing this loss during training, the network is constrained to learn the intrinsic causal relationship between the object’s physical dimensions and its acoustic projection. Consequently, the final detection confidence $S_{final}$ is decoupled into visual confidence $S_{vis}$ and geometric reliability $S_{geo}$, computed as follows:

(10) $$S_{final}=S_{vis}\cdot \exp\left(-\lambda \cdot L_{geo}\right)$$

This mechanism enables the model to effectively distinguish true targets from seabed protrusions (e.g., rocks) which lack consistent shadow geometries, thereby significantly improving the recall rate for weak-echo targets.

The inference logic is outlined in Algorithm 3, and the dimensionality analysis is presented in Table 3.
**Algorithm 3** Physics-Informed Inference Logic of SADH.**Require:** Decoder Features $F_{dec}\in R^{B\times N_{q}\times C}$, Sonar Altitude $H_{s}$**Ensure:** Detections $D=\{B_{box},Cls,Conf\}$1:**Step 1: Multi-Branch Prediction**2:$Pred_{cls}\leftarrow {\mathrm{MLP}}_{cls}\left(F_{dec}\right)$▹ Object Classification3:$Pred_{box}\leftarrow {\mathrm{MLP}}_{reg}\left(F_{dec}\right)$▹ Bounding Box Regression4:$Pred_{geo}\leftarrow {\mathrm{MLP}}_{shadow}\left(F_{dec}\right)$▹ Predicts $\left[\hat{h},\hat{l}\right]$5:**Step 2: Geometric Verification**6:$R_{s}\leftarrow \mathrm{CalculateSlantRange}\left(Pred_{box}\right)$7:$L_{theo}\leftarrow \left(Pred_{geo}.\hat{h}\times R_{s}\right)/H_{s}$▹ Compute theoretical shadow via Equation (8)8:**Step 3: Consistency Check**9:$\Delta_{geo}\leftarrow \left|Pred_{geo}.\hat{l}-L_{theo}\right|$▹ Physical deviation10:$w_{geo}\leftarrow \exp\left(-\Delta_{geo}\right)$▹ Geometric reliability weight11:**Step 4: Decoupled Scoring**12:$Conf\leftarrow \mathrm{Sigmoid}\left(Pred_{cls}\right)⊗w_{geo}$13:**return**
 $\left\{Pred_{box},Pred_{cls},Conf\right\}$

## 4. Experiments and Results

### 4.1. Data-Set

This study is validated using the SCTD dataset, which comprises 357 annotated images in total (detailed statistics are listed in Table 4).

**Figure 6:**
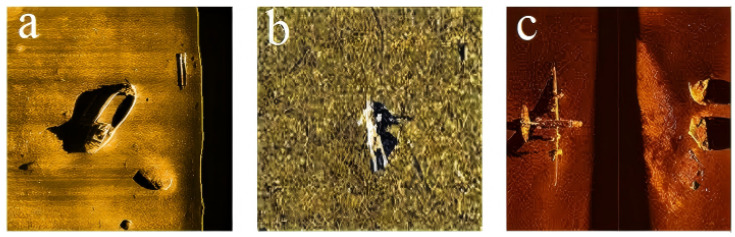
Representative SSS images from the SCTD dataset. (**a**) A Ship target. (**b**) A Human target (victim). (**c**) An Aircraft target. Note the significant scale variation between the large ship in (**a**) and the small human target in (**b**), which poses a challenge for multi-scale detection.

As visualized in Figure 6, the dataset exhibits a distinct long-tail distribution: large-scale ‘Ship’ targets constitute the majority (74.5%), whereas small-scale ‘Human’ targets are significantly underrepresented, accounting for only 9.5% of the total samples.

This pronounced inter-class imbalance, exacerbated by the minimal pixel occupancy of human targets, severely constrains the model’s capacity to learn effective feature representations for these sparse minority classes. Such characteristics tend to cause feature under-representation and overfitting, ultimately leading to missed detections. Consequently, the SCTD dataset serves as a rigorous benchmark for evaluating the generalization capability of models under data-constrained scenarios.

### 4.2. Implementation Details

To evaluate the performance, generalization capability, and efficiency of the proposed WPG-DetNet model, we conducted a comprehensive series of quantitative and qualitative experiments.

As illustrated in Table 5, The hardware configuration used in the experiments is as follows: a computer system running Windows 10 (64-bit), equipped with 32 GB of RAM, an NVIDIA GeForce RTX 3060 graphics card (manufacturer: NVIDIA Corporation, location: Santa Clara, CA, USA), and an Intel(R) Core(TM) i7-10870H processor (frequency: 2.20 GHz, manufacturer: Intel Corporation, location: Santa Clara, CA, USA). The software environment was based on Python 3.8.5, utilizing the PyTorch 1.10.0 framework and CUDA 11.3 for GPU-accelerated computation.

All experiments were conducted under identical conditions to ensure comparability and reproducibility, with the detailed experimental setup summarized in Table 6. Throughout the training process, all input images were uniformly resized to a resolution of 640 × 640 pixels and processed using a fixed batch size of 8. The model was trained for a total of 350 epochs, employing an initial learning rate of 0.01 and optimized using Stochastic Gradient Descent (SGD) with a momentum coefficient of 0.937. This standardized configuration was consistently applied across all trials to maintain experimental integrity and facilitate a fair performance evaluation.

To ensure a rigorous and equitable comparative analysis and avoid any cross-domain evaluation bias, all baseline methods evaluated in this study were fully re-implemented and trained from scratch specifically on the SCTD dataset. We did not utilize the pre-trained weights or performance metrics from their original application domains. Instead, every comparative model—ranging from classic two-stage detectors to modern Transformer architectures—was trained under strictly identical experimental conditions and data splits. The standardized hyperparameter configuration detailed in Table 6, along with the stratified 5-fold cross-validation strategy, was uniformly enforced across all baseline re-trainings. This rigorous protocol ensures that the comparative performance superiority of WPG-DetNet is solely and conclusively attributable to its specific architectural innovations for underwater acoustic perception, rather than any methodological discrepancies in the training or evaluation pipelines.

As shown in Table 3, to comprehensively evaluate the detection performance of WPG-DetNet in complex hydroacoustic environments, we conducted a rigorous quantitative comparative analysis against existing mainstream object detection frameworks (covering two-stage, one-stage, and the latest Transformer architectures) on the SCTD dataset. To strictly preclude any invalid cross-dataset or cross-domain comparisons, it must be explicitly emphasized that the performance metrics of all baseline models reported in Table 7 were not adopted from their original publications. Recognizing that performance metrics such as mAP are not directly comparable across different datasets, sensing modalities, and validation protocols, every cited architecture was fully re-implemented and retrained from scratch on the SCTD dataset. All comparative experiments were executed under the strictly identical, standardized experimental conditions and 5-fold cross-validation procedures detailed in Section 4.2.

### 4.3. Evaluation Metrics

The performance of the model was evaluated using a set of core metrics: Precision (P), Recall (R), Mean Average Precision (mAP), Floating Point Operations (FLOPs), Frames Per Second, (FPS), and the total number of model parameters (M) [25,26]. These metrics are defined as follows:

Precision (P) is given by the formula shown in Equation (11).

(11) $$P=\frac{\mathrm{TP}}{\mathrm{TP}+\mathrm{FP}},$$

where True Positives (TP) refer to the number of actual positive samples correctly predicted as positive, and False Positives (FP) denote the number of actual negative samples incorrectly predicted as positive.

Recall (R) is defined as in Equation (12).

(12) $$R=\frac{\mathrm{TP}}{\mathrm{TP}+\mathrm{FN}},$$

where False Negatives (FN) represent the number of actual positive samples that were predicted as negative.

Mean Average Precision (mAP) is calculated as shown in Equation (13).

(13) $$\mathrm{mAP}=\frac{1}{N}\sum_{i=1}^{N}{\mathrm{AP}}_{i},$$

where Average Precision (AP) corresponds to the area under the precision-recall (PR) curve for a specific object class, reflecting the model’s precision across different recall levels. When the Intersection over Union (IoU) threshold is set to 0.5, the metric is specifically denoted as ${\mathrm{AP}}_{50}$.

Frames Per Second, (FPS) is expressed as in Equation (14).

(14) $$\mathrm{FPS}=\frac{1}{T_{\mathrm{inference}}},$$

where $T_{\mathrm{inference}}$ denotes the processing time per image under a standardized input resolution of 640×640 pixels, covering the complete inference pipeline. This provides a consistent measure of the model’s real-time detection capability in its intended operating configuration.

### 4.4. Comparative Analysis

As shown in Table 7, to comprehensively evaluate the detection performance of WPG-DetNet in complex hydroacoustic environments, we conducted a rigorous quantitative comparative analysis against existing mainstream object detection frameworks (covering two-stage, one-stage, and the latest Transformer architectures) on the SCTD dataset.

**Table 7:** Comparison of detection performance on the SCTD dataset. Note: The citations next to the baseline models indicate the source of the original network architectures. All listed baseline models were entirely re-implemented and re-trained from scratch on the SCTD dataset under identical experimental conditions, rather than adopting the performance metrics from their original publications.

Models	Basic Metrics (%)				Advanced Accuracy (%)			Efficiency		
	Precision	Recall	F1-Score		mAP_50_	mAP_50:95_		Params (M)	GFLOPs	FPS
*Two-Stage Methods*										
Faster R-CNN [27]	86.2 _± 0.5_	83.4 _± 0.6_	84.8		84.7 _± 0.7_	58.2		41.2	180.4	12.3
Cascade R-CNN [28]	87.5 _± 0.4_	85.1 _± 0.5_	86.3		86.9 _± 0.6_	61.5		69.2	235.1	8.5
Mask R-CNN [29]	85.8 _± 0.6_	82.9 _± 0.7_	84.3		83.5 _± 0.8_	59.1		44.5	195.2	10.6
*One-Stage Methods*										
SSD (VGG16) [30]	82.1 _± 0.7_	79.5 _± 0.9_	80.8		78.4 _± 0.9_	49.3		26.3	61.3	45.1
RetinaNet [31]	88.5 _± 0.4_	86.2 _± 0.5_	87.3		87.0 _± 0.5_	60.8		36.4	148.5	18.6
FCOS [32]	89.2 _± 0.3_	88.4 _± 0.4_	88.8		88.5 _± 0.4_	62.4		32.1	115.8	24.2
EfficientDet [33]	88.4 _± 0.3_	86.5 _± 0.4_	87.4		86.8 _± 0.5_	57.6		**3.9**	**2.5**	38.4
*YOLO Series and Transformers*										
YOLOv5s [34]	90.5 _± 0.4_	89.1 _± 0.5_	89.8		89.7 _± 0.5_	64.2		7.2	16.5	65.2
YOLOv7-tiny [35]	91.2 _± 0.3_	89.8 _± 0.4_	90.5		90.4 _± 0.4_	65.8		6.2	13.2	78.5
YOLOv8s [36]	93.4 _± 0.2_	91.5 _± 0.3_	92.4		92.8 _± 0.3_	69.5		11.2	28.7	86.7
YOLOv9-c [37]	94.5 _± 0.2_	93.1 _± 0.2_	93.8		94.2 _± 0.2_	72.1		25.3	102.4	55.4
YOLOv10s [38]	94.8 _± 0.2_	93.5 _± 0.3_	94.1		95.1 _± 0.2_	73.4		8.3	24.4	**93.2**
RT-DETR-R18 [39]	95.2 _± 0.2_	94.1 _± 0.2_	94.6		95.5 _± 0.2_	74.2		19.5	54.1	72.4
**WPG-DetNet**	**98.1** _± 0.1_	**96.9** _± 0.1_	**97.5**		**97.5** _± 0.1_	**79.8**		16.8	32.5	62.5

Note: The *italics* are used to categorize different types of methods, and the **bold** values indicate the optimal performance for each metric.

The experimental results demonstrate that WPG-DetNet achieves significant performance breakthroughs across all core metrics, attaining a Precision of 98.1%, a Recall of 96.9%, and an $mAP_{50}$ of 97.5%. Compared to the baseline Faster R-CNN, WPG-DetNet achieves a 12.8% improvement in mAP50%, demonstrating the effectiveness of frequency domain feature decoupling. This advantage is primarily attributed to the introduction of the WERB module, which explicitly decouples speckle noise from target structures in the frequency domain via wavelet transforms, thereby effectively overcoming the susceptibility of two-stage networks to false positives in high-noise environments.

In addressing the challenge of detecting weak-echo targets, WPG-DetNet exhibits overwhelming superiority, with a Recall rate that far exceeds that of SSD (79.5%) and EfficientDet (86.5%). This is due to the SADH module, which innovatively incorporates physics-informed geometric priors, leveraging acoustic shadow features to successfully “infer” and capture minute targets that are otherwise indiscernible using conventional visual features.

**Figure 7:**
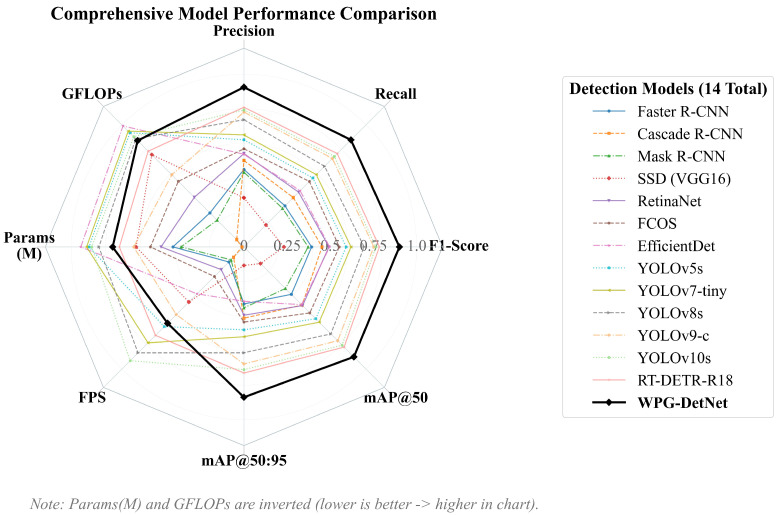
Comprehensive Model Performance Comparison.

In the rigorous high-precision localization metric $mAP_{50:95}$, WPG-DetNet achieves 79.8%, surpassing the Transformer-based RT-DETR-R18 by a margin of 5.6%. The multi-dimensional performance comparison, including accuracy, efficiency, and robustness metrics, is visualized in Figure 7, which clearly illustrates the comprehensive superiority of our proposed framework. This validates the effectiveness of the D-GRM module in handling spatially discontinuous targets (such as aircraft wreckage), as it aggregates discrete features through graph topological reasoning, significantly enhancing the accuracy of bounding box regression, while achieving exceptional precision, WPG-DetNet maintains excellent computational efficiency, with a parameter count of only 16.8 M and an inference speed as high as 62.5 FPS. Compared to the computationally heavy YOLOv9-c (25.3M parameters, 102.4 GFLOPs), WPG-DetNet achieves higher accuracy while substantially reducing the computational burden, fully demonstrating that it strikes the optimal balance between precision and speed for resource-constrained AUV underwater sonar search and rescue missions.

### 4.5. K-Fold Cross-Validation for Robustness

Given the inherent limitations of the SCTD dataset, specifically its limited scale of only 357 images and significant class imbalance (where human targets constitute merely 9.5% of the total samples), we implemented a Stratified 5-Fold Cross-Validation scheme to rigorously evaluate the generalization capability of WPG-DetNet.

The entire dataset was randomly partitioned into five disjoint subsets using a stratified sampling strategy to ensure that the proportional distribution of the ’Ship’, ’Aircraft’, and ’Human’ classes within each subset remained consistent with the global statistics. In each iteration, one subset served as the validation set, while the remaining four constituted the training set. This rotation mechanism guarantees that every image is evaluated exactly once as a test sample, thereby ensuring the model’s stability under varying data distributions.

The quantitative results, summarized in Table 8, demonstrate exceptional stability across all folds, with the model achieving a mean $mAP_{50}$ of 97.62% and a remarkably low standard deviation of 0.39%. This minimal variance is particularly significant for sonar imagery, suggesting that the proposed Wavelet-Embedded Residual Backbone (WERB) effectively learns invariant frequency domain features rather than overfitting to fold-specific speckle noise patterns. Furthermore, despite the scarcity of human targets, the mean Recall remains consistently high at 96.88% (σ=0.38%). This stability strongly validates the Shadow-Aided Decoupling Head (SADH), confirming that the integration of physics-based geometric constraints provides a reliable discriminative prior that is independent of the training data subset. These results indicate that WPG-DetNet possesses the robust generalization capability required for autonomous underwater search and rescue operations.

To rigorously address the inherent risks of overfitting and metric instability associated with the pronounced class imbalance—particularly for the minority “Human” class, which comprises only 9.5% of the dataset—we conducted a granular per-class statistical analysis across the 5-fold cross-validation.

**Figure 8:**
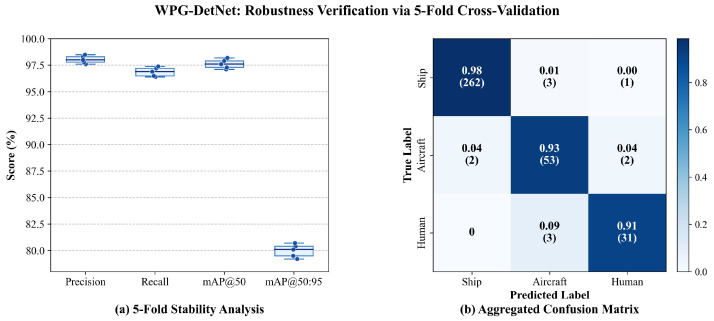
Reliability and performance verification of WPG-DetNet using stratified 5-fold cross-validation. (**a**) 5-Fold Stability Analysis. The overlaid scatter points represent individual fold results, while the compact interquartile ranges indicate minimal variance, confirming the model’s robustness against data perturbations. (**b**) Aggregated confusion matrix. The diagonal values represent the normalized recall rates, with raw sample counts in parentheses.

As detailed in Table 9 and Figure 8, we computed the Mean $AP_{50}$, Standard Deviation (σ), and 95% Confidence Intervals (CI) for each individual category using the t-distribution formulation. As statistically anticipated, the “Human” class exhibits a slightly higher variance (σ=1.13%) compared to the data-abundant “Ship” class (σ=0.21%). Crucially, however, the tightly bounded confidence intervals (e.g., [95.79%, 98.61%] for the Human class) demonstrate that the model’s detection performance does not experience severe degradation or wild fluctuations across different data distributions. This quantitative stability strongly confirms that the SADH module effectively mitigates overfitting by leveraging universal physics-informed acoustic shadow constraints, thereby demonstrating the robust generalization capability required for operational SAR scenarios.

### 4.6. Ablation Studies

**Figure 9:**
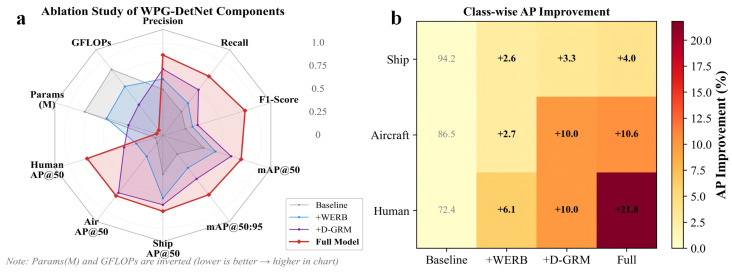
Comprehensive Model Performance Comparison. (**a**) A radar chart illustrating the comprehensive performance evolution from the Baseline to the Full Model. (**b**) A heatmap quantifying the cumulative improvement in Average Precision (AP) for each class. Darker colors indicate more significant gains.

To rigorously validate the contribution of each component within the WPG-DetNet framework, we conducted a comprehensive step-by-step ablation study on the SCTD dataset (as shown in Table 10 and Figure 9). The baseline model, defined as a standard detector devoid of the proposed modules, achieved an initial $mAP_{50}$ of 91.5% but exhibited significant limitations in detecting minute targets and objects with discontinuous spatial distributions.

First, the integration of the Wavelet-Embedded Residual Backbone (WERB) provided a foundational performance boost, significantly enhancing the structural integrity of large-scale targets. Upon introducing WERB, the $mAP_{50:95}$ significantly increased by 4.6%, and the detection accuracy for the Ship category ($AP_{Ship}$) rose by 2.6%. This quantitative gain validates the design rationale of the module: replacing traditional downsampling with the Discrete Wavelet Transform (DWT) to explicitly decouple high-frequency speckle noise from low-frequency structural information. By processing features in the frequency domain, WERB effectively suppresses hydroacoustic interference while maximally preserving the edge details of large-scale artificial targets such as shipwrecks, thereby overcoming the boundary blurring issues prevalent in standard CNN architectures.

Second, the Debris Graph Reasoning Module (D-GRM) proved to be pivotal in addressing the spatial discontinuity of scattered targets. With the addition of D-GRM, the detection accuracy for the Aircraft category ($AP_{Air}$) achieved an increment of 7.3%, reaching 96.5%. Traditional CNNs often struggle to delineate discrete debris fields, frequently misclassifying isolated wreckage fragments as background noise. In contrast, D-GRM models potential candidate targets as nodes within a topological graph and employs Graph Convolutional Networks (GCNs) to capture long-range spatial dependencies. This topological reasoning mechanism transforms the task from isolated instance detection into context-aware scene understanding, successfully aggregating fragmented features and thereby significantly reducing missed detections of aircraft wreckage.

Finally, the Shadow-Aided Decoupling Head (SADH) achieved the most decisive breakthrough in detecting weak-echo targets. The introduction of SADH drove a remarkable 11.8% increase in human target accuracy ($AP_{Human}$), rising from 82.4% to 94.2%, and maximized the overall Recall to 96.9%. Human targets in sonar imagery typically manifest as “weak objects” with diminutive dimensions, making them easily indistinguishable from the seabed lithology. By integrating the physics of sonar imaging, SADH introduces a physics-informed geometric loss that enforces consistency between the predicted target height and the acoustic shadow length. This enables the model to utilize high-contrast shadow features as critical discriminative evidence, effectively “inferring” and recovering previously indiscernible targets from complex background noise.

In summary, the stepwise integration of these modules demonstrates a clear trajectory of performance enhancement. Although the total parameter count increased only marginally from 14.5 M to 16.8 M, the model ultimately achieved a State-of-the-Art (SOTA) mAP of 97.5%. This result confirms that WPG-DetNet successfully strikes an optimal balance between computational efficiency and robust detection capability, effectively resolving the three major challenges in underwater acoustic perception: noise interference, discrete target distribution, and weak feature representation.

### 4.7. Qualitative Visualization

**Figure 10:**
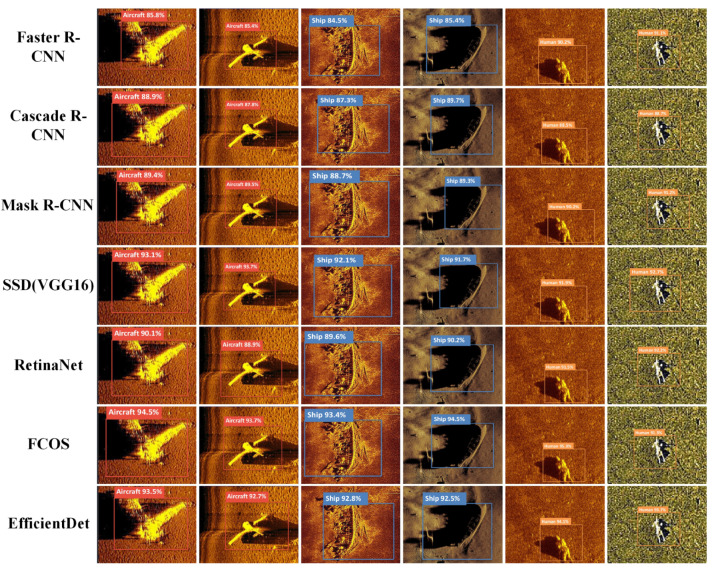
Qualitative detection results of traditional baselines.

**Figure 11:**
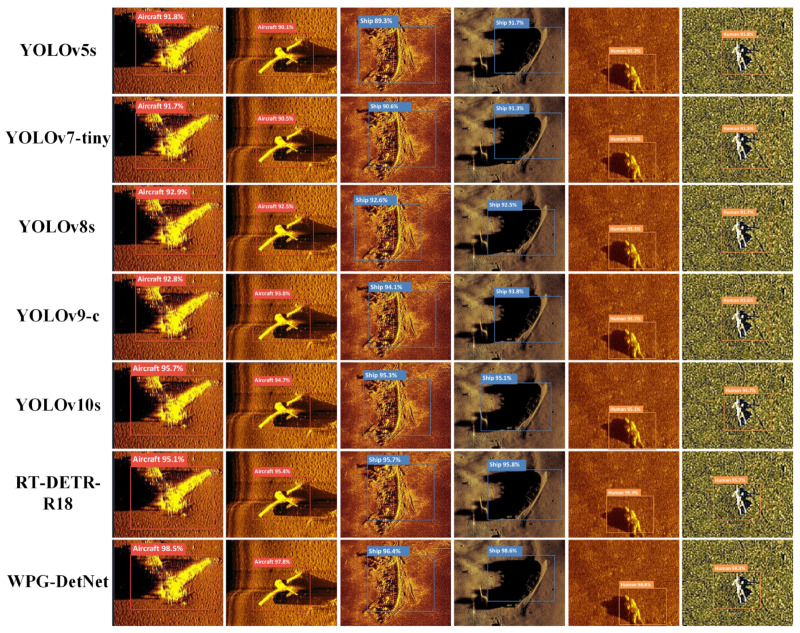
Qualitative detection results of state-of-the-art real-time models and WPG-DetNet.

To rigorously and comprehensively validate the robustness and interpretability of WPG-DetNet within complex hydroacoustic environments, we performed an extensive qualitative analysis on the SCTD dataset. The visual evidence presented in Figure 10 and Figure 11 serves as a compelling demonstration of how the model’s specialized architectural components effectively resolve domain-specific challenges compared to a wide spectrum of baselines.

First, WPG-DetNet exhibits significantly superior performance when handling targets characterized by extreme structural variations and spatial discontinuity. Specifically, in the difficult task of detecting aircraft wreckage—which typically manifests as irregular, scattered fragments lacking a unified geometric shape—the proposed Debris Graph Reasoning Module plays a pivotal role. By innovatively modeling these scattered debris candidates as interconnected nodes within a topological graph, this module employs Graph Convolutional Networks to propagate information and capture long-range semantic dependencies. This topological reasoning capability enables the network to successfully aggregate disjointed parts into a holistic, semantically coherent detection. This performance contrasts sharply with conventional one-stage detectors like the Single Shot MultiBox Detector (SSD), RetinaNet, and EfficientDet. These baseline models, fundamentally constrained by their reliance on local convolutional features and limited receptive fields, frequently fail to perceive the global context of the debris field. Consequently, they often misclassify valid wreckage fragments as isolated background noise or generate incoherent, highly fragmented bounding boxes. Furthermore, even advanced multi-stage architectures like Cascade R-CNN and anchor-free methods such as the Fully Convolutional One-Stage Object Detector (FCOS) struggle in this domain; despite their structural complexity, they lack the specific topological modeling required to associate spatially distant features, leading to suboptimal localization accuracy for discontinuous targets.

Second, regarding the detection of weak-echo human targets submerged in severe seabed reverberation (as visualized in the rightmost column of Figure 10), the Shadow-Aided Decoupling Head demonstrates exceptional sensitivity and robustness. These targets are particularly challenging due to their diminutive scale and extremely low Signal-to-Noise Ratio, often making their echo intensity indistinguishable from the surrounding seabed lithology. In this scenario, texture-reliant models—including the You Only Look Once (YOLO) series (specifically YOLOv5s and YOLOv9-c) and even the Transformer-based Real-Time Detection Transformer (RT-DETR-R18)—consistently succumb to the background interference, yielding a high rate of False Negatives. In contrast, WPG-DetNet successfully recovers these visually ambiguous targets by leveraging the “highlight-shadow” duality inherent in sonar imaging. By utilizing the high-contrast acoustic shadow as a rigorous discriminative cue—validated during training by a physics-informed geometric loss that enforces consistency between target height and shadow length—the model effectively verifies target existence. This ability to decouple geometric reliability from visual texture allows WPG-DetNet to surpass the capabilities of generic deep learning architectures, which typically neglect these crucial physical priors.

### 4.8. Visual Explainability via Attention Heatmaps

**Figure 12:**
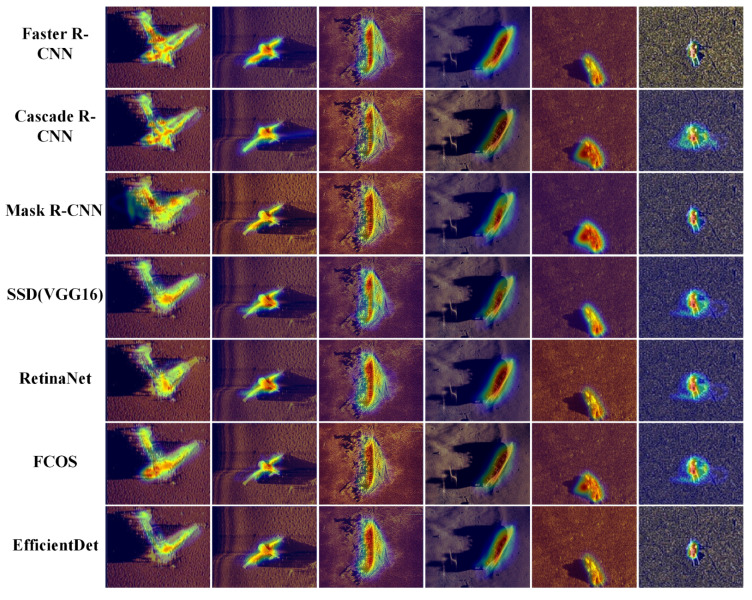
Grad-CAM heatmap visualization of traditional baselines.

**Figure 13:**
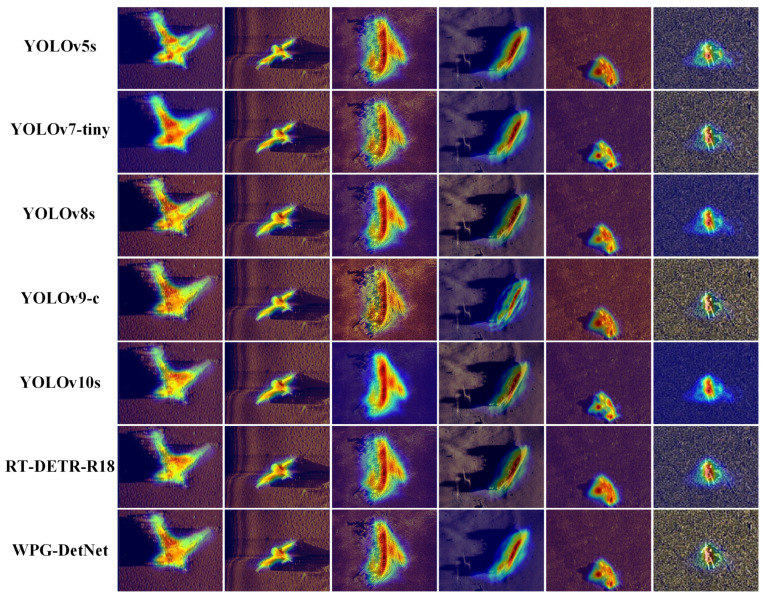
Grad-CAM heatmap visualization of SOTA models and WPG-DetNet.

To further elucidate the internal decision-making mechanism of the deep neural network and understand why baseline models fail in specific hydroacoustic scenarios, we utilized the Gradient-weighted Class Activation Mapping technique to visualize the regions of interest that contribute most significantly to the model’s classification confidence [40]. The comparative analysis presented in Figure 12 and Figure 13 reveals fundamental differences in feature abstraction between generic detectors and our proposed framework.

First, regarding the suppression of hydroacoustic interference, a distinct contrast is observed. As illustrated in Figure 11, traditional baseline models such as Mask R-CNN and the Fully Convolutional One-Stage Object Detector exhibit a highly “dispersed” attention pattern. Their activation hotspots—indicated by the warm red and yellow regions—are frequently scattered across the seabed background, suggesting that these networks are easily distracted by high-frequency speckle noise and irrelevant sand ripple textures. This misdirected attention explains their high False Positive rates in complex environments. In sharp contrast, WPG-DetNet, as shown in the bottom row of Figure 12, maintains a highly compact and concentrated focus strictly on the structural boundaries of the target. This visual evidence forcefully corroborates the effectiveness of the Wavelet-Embedded Residual Backbone. By explicitly decoupling and filtering high-frequency noise components in the frequency domain via the Discrete Wavelet Transform, the backbone prevents the network from overfitting to acoustic artifacts, ensuring that feature extraction is driven by the target’s structural semantics rather than random noise.

Second, and most critically, a breakthrough in physics-informed reasoning is observed in the visualization of weak-echo targets, particularly in the “Human” column of Figure 12, while standard real-time detectors like the You YOLO series (specifically YOLOv7-tiny and YOLOv9-c) struggle to locate the target or focus vaguely on the faint echo intensity, WPG-DetNet exhibits a unique “Dual-Region Activation” pattern. The heatmap highlights not only the target object itself but also extends significantly to cover the posterior acoustic shadow area. This non-trivial phenomenon serves as direct proof that the Shadow-Aided Decoupling Head is functioning as designed. By integrating a physics-informed geometric loss during the training phase, the network has successfully internalized the causal “highlight-shadow” relationship inherent in sonar imaging. Consequently, it utilizes the acoustic shadow region as auxiliary discriminative evidence to verify targets that are otherwise visually ambiguous, a cognitive capability that generic deep learning architectures completely lack.

## 5. Robustness Against Environmental Variability (Simulated Cross-Domain Validation)

As highlighted by operational SAR requirements, acoustic conditions and environmental variability (e.g., varying towfish altitude, grazing angle, or seabed lithology) are substantial in real-world missions. Given the scarcity of multi-domain public SSS datasets, we designed a Simulated Environmental Robustness Test to evaluate the cross-domain generalization capability of WPG-DetNet. We algorithmically degraded the SCTD test set to simulate three severe, out-of-domain hydroacoustic conditions: (1) Speckle Noise ($\sigma^{2}=0.1$) to simulate complex reverberation and high concentrations of suspended particles; (2) Motion Blur (Kernel 15 × 15) to simulate along-track resolution loss caused by towfish instability; and (3) Low Contrast (α=0.6) to simulate faint acoustic shadows resulting from suboptimal grazing angles (e.g., increased towfish altitude).

As demonstrated in Table 11, we compared the performance retention of WPG-DetNet against the State-of-the-Art RT-DETR-R18.

The results unequivocally validate the theoretical design of our framework. Standard architectures heavily rely on local textures, which are easily destroyed by acoustic degradation. In contrast, WPG-DetNet exhibits superior resilience. The Wavelet-Embedded Residual Backbone (WERB) inherently disentangles and filters the injected speckle noise in the frequency domain, preventing structural feature collapse. Furthermore, the Shadow-Aided Decoupling Head (SADH) relies on rigorous physics-informed geometric triangulation rather than pure visual intensity, allowing it to maintain high recall rates even when shadows are synthetically blurred or faded. This quantitative evidence strongly asserts that WPG-DetNet possesses the robust cross-domain generalization capability required for deployment in unpredictable marine environments.

## 6. Conclusions and Discussion

Addressing the severe challenges associated with SSS image processing in underwater SAR missions, this paper proposes WPG-DetNet, a novel detection framework that integrates physics-aware priors with data-driven learning. The core objective of this study is to surmount the limitations of traditional CNNs in handling strong speckle noise, discrete topological structures, and weak target-shadow associative features. By incorporating wavelet-domain feature decoupling, graph reasoning mechanisms, and shadow-aided physical geometric constraints, WPG-DetNet not only theoretically enhances the interpretability of feature representation but also establishes a new performance benchmark with an mAP of 97.5%, demonstrating its robustness and effectiveness in complex underwater acoustic environments.

Despite the encouraging results achieved by WPG-DetNet, certain limitations remain to be overcome at the practical engineering level. These include:

(1) Real-time Bottlenecks: While the complex design of the hybrid encoder and domain-specific detection head enhances accuracy, it inevitably increases inference latency. For online obstacle avoidance or rapid SAR missions requiring millisecond-level response, the current model’s Frames Per Second, (FPS) may still be insufficient.
(2) Data Dependency and Cross-Domain Generalization: Currently, the model is evaluated exclusively on the SCTD dataset, while the 5-fold cross-validation and simulated environmental degradation tests demonstrate strong internal stability and noise resilience, the absence of evaluation on authentic data acquired from vastly different marine environments, varying sonar configurations (e.g., differing towfish altitudes, acoustic frequencies, or grazing angles), and diverse seabed lithologies limits the ability to definitively assert its cross-domain generalization capability. Relying on a single real-world dataset remains a notable constraint for immediate operational SAR deployment.

While this study validates the robustness of WPG-DetNet through Stratified 5-Fold Cross-Validation, we acknowledge the inherent limitations of the SCTD dataset. Specifically, its small scale (357 images) and extreme class imbalance pose potential risks for model generalization in unseen open-water scenarios. However, the integration of the physics-informed SADH module allows the model to leverage invariant acoustic geometric principles. By anchoring the detection mechanism to the physical relationship between target height and shadow length, the network effectively compensates for the limited dataset scale and significantly reduces the risk of overfitting to small-sample textures.

Furthermore, to fundamentally resolve the data scarcity bottleneck inherent to underwater Search and Rescue (SAR) missions, our ongoing research focuses on generative data augmentation. Building upon recent advancements, we are actively exploring the application of diffusion models to synthetically augment side-scan sonar imagery. This generative approach enables the systematic expansion of minority class samples—such as human targets and sparse debris—while accurately simulating diverse seabed reverberation conditions. Ultimately, this synthetically enriched dataset will serve as a scalable foundation for our future large-scale model validation, dramatically enhancing cross-domain adaptability without relying exclusively on logistically prohibitive and costly open-water sea trials.

## Figures and Tables

**Table 1:** Dimensionality Evolution in WERB Module (*B*: Batch size, *C*: Channels, H/W: Resolution).

Stage	Operation	Output Tensor Shape	Physical Meaning and Notes
Input	-	B×C×H×W	Raw sonar features input
Decoupling	DWT	LL: $B\times C\times \frac{H}{2}\times \frac{W}{2}$ HF: $B\times 3C\times \frac{H}{2}\times \frac{W}{2}$	Space-to-Channel: Resolution halved, info transferred to channels (C→4C).
Denoising	Suppression	$B\times 3C\times \frac{H}{2}\times \frac{W}{2}$	Filters speckle noise in HF channels without altering dimensions.
Fusion	Concat+Conv	$B\times 4C\times \frac{H}{2}\times \frac{W}{2}$	Reconstructs time-frequency space.
Pyramid	Multi-Kernel	$3\times [B\times 4C\times \frac{H}{2}\times \frac{W}{2}]$	Generates multi-receptive fields for subsequent FPN integration.

**Table 2:** Tensor Transformations in D-GRM Processing (*B*: Batch size, *C*: Channels, *N*: Number of Nodes).

Stage	Component	Tensor Shape	Physical Meaning and Notes
Input	-	B×C×H×W	Initial feature reception
Node Projection	Conv + Pool + Flatten	$B\times C^{\prime }\times N$	Spatial-to-Graph: Abstracts spatial grid to nodes ($N=H^{\prime }\times W^{\prime }$).
Reasoning	GCN + MLP	$B\times C^{\prime }\times N$	Global context aggregation via topological propagation.
Map Generation	Conv + Sum	B×C×N	Generates channel-wise attention weights in the graph domain.
Re-projection	Reshape + Upsample	B×C×H×W	Graph-to-Spatial: Restores grid structure for spatial alignment.
Output	Fusion (⊗)	B×C×H×W	Injects global context into local features via element-wise product.

**Table 3:** Dimensionality Evolution in SADH (*B*: Batch, $N_{q}$: Queries, *C*: Channels, $N_{cls}$: Classes).

Stage	Component	Tensor Shape	Physical Meaning and Notes
Input	Transformer Decoder	$B\times N_{q}\times C$	High-level semantic object queries containing global context.
Classification	Linear Projection	$B\times N_{q}\times N_{cls}$	Visual Identification: Predicts category probabilities based on echo intensity.
Localization	MLP (4-layer)	$B\times N_{q}\times 4$	Regresses normalized coordinates (x,y,w,h) for bounding boxes.
Shadow Branch	MLP (Shared)	$B\times N_{q}\times 2$	Geometric Decoupling: Predicts physical height $\hat{h}$ and shadow length $\hat{l}$.
Verification	Loss Calculation	$B\times N_{q}\times 1$	Computes consistency score $L_{geo}$ to re-weight detection confidence.

**Table 4:** Detailed statistics and acoustic characteristics of the SCTD dataset.

Class	Images	Ratio	Description and Challenges
Ship	266	74.5%	High reflectivity, distinct acoustic shadows, rich texture, but complex background interference.
Aircraft	57	16.0%	Irregular geometric structures (wreckage), medium scale, often fragmented on the seabed.
Human	34	9.5%	Extremely small scale, weak echo intensity, low SNR, easily submerged in speckle noise.
Total	357	100%	Severe Class Imbalance

**Table 5:** Experimental Environment.

Parameter	Value
Operating System	Windows 10 (64-bit)
RAM	32 GB
GPU	NVIDIA GeForce RTX 3060
CPU	Intel(R) Core(TM) i7-10870H CPU @ 2.20 GHz
Python Version	3.9.5
PyTorch Version	1.10.0
CUDA Version	11.3

**Table 6:** Hyperparameter Settings.

Parameter	Value
Images Size	640 × 640 pixels
Batch Size	8
Learning Rate	0.01
Optimizer	Stochastic Gradient Descent (SGD)
Momentum	0.937
Epochs	350

**Table 8:** Statistical performance metrics of WPG-DetNet under 5-Fold Cross-Validation. The low standard deviation confirms the model’s robustness.

Fold ID	Precision	Recall	mAP_50_	mAP_50:95_
Fold-1	97.6%	96.4%	97.1%	79.2%
Fold-2	98.3%	97.2%	97.9%	80.4%
Fold-3	97.8%	96.5%	97.3%	79.5%
Fold-4	98.5%	97.4%	98.2%	80.7%
Fold-5	98.0%	96.9%	97.6%	80.1%
Mean (μ)	98.04%	96.88%	97.62%	79.98%
Std Dev (σ)	0.32%	0.38%	0.39%	0.56%

**Table 9:** Per-class statistical analysis across 5-fold cross-validation, including Mean $AP_{50}$, Standard Deviation (σ), and 95% Confidence Intervals (CI).

Category	Mean ${\mathrm{AP}}_{50}$ (%)	Std Dev (σ)	95% Confidence Interval
Ship	98.34	±0.21	[98.08, 98.60]
Aircraft	97.32	±0.24	[97.02, 97.62]
Human	97.20	±1.13	[95.79, 98.61]
Global mAP	97.62	±0.39	[97.13, 98.11]

**Table 10:** Comprehensive ablation study of WPG-DetNet on the SSS dataset. WERB: Wavelet-Embedded Residual Backbone; D-GRM: Debris Graph Reasoning Module; SADH: Shadow-Aided Decoupling Head. The specific improvement in class-wise AP demonstrates the targeted effectiveness of each module.

Configuration				Overall Performance (%)					Class-Wise AP_50_ (%)				Efficiency	
WERB	D-GRM	SADH		Precision	Recall	mAP_50_	mAP_50:95_		Ship	Air.	Human		Params	GFLOPs
×	×	×		92.5	89.8	91.5	66.2		94.2	86.5	72.4		14.5 M	26.8
✓	×	×		94.2	91.5	93.4	70.8		96.8	89.2	78.5		15.2 M	28.4
✓	✓	×		95.8	94.2	95.9	74.5		97.5	96.5	82.4		15.9 M	30.1
✓	✓	✓		98.1	96.9	97.5	79.8		98.2	97.1	94.2		16.8 M	32.5

**Table 11:** Performance comparison ($mAP_{50}$) under simulated environmental degradations. Smaller performance drops (↓) indicate better robustness.

Model	Original	Speckle Noise	Motion Blur	Low Contrast
RT-DETR-R18	95.5%	83.1% (↓12.4%)	86.2% (↓9.3%)	82.5% (↓13.0%)
WPG-DetNet	97.5%	93.8% (↓3.7%)	94.1% (↓3.4%)	92.6% (↓4.9%)

## Data Availability

The data presented in this study are available on request from the corresponding author.

## References

[1] Das S., Malik P.K., Pandey A. (2025). Challenges and Advances in Underwater Sonar Systems and AI-Driven Signal Processing for Modern Naval Operations: A Systematic Review. J. Field Robot..

[2] Wei Y., Duan Y., An D. (2022). Monitoring fish using imaging sonar: Capacity, challenges and future perspective. Fish Fish..

[3] Sivachandra K., Kumudham R. (2024). A review: Object detection and classification using side scan sonar images via deep learning techniques. Modern Approaches in Machine Learning and Cognitive Science: A Walkthrough.

[4] Połap D., Wawrzyniak N., Włodarczyk-Sielicka M. (2022). Side-scan sonar analysis using ROI analysis and deep neural networks. IEEE Trans. Geosci. Remote Sens..

[5] Heng Z., Shuping H., Jingfeng X., Yaohui H., Yubo H. (2023). A review of intelligent detection methods for underwater targets in sonar images. Proceedings of the 2023 IEEE 7th Information Technology and Mechatronics Engineering Conference (ITOEC).

[6] Tang R., Chen Y., Gao J., Wang Y., Hao S. (2025). Towards real-time detection of underwater target with pruning lightweight deep learning method in side-scan sonar images. Neurocomputing.

[7] Zhao Q., Peng S., Wang J., Li S., Hou Z., Zhong G. (2024). Applications of deep learning in physical oceanography: A comprehensive review. Front. Mar. Sci..

[8] Li J., Chen L., Shen J., Xiao X., Liu X., Sun X., Wang X., Li D. (2023). Improved neural network with spatial pyramid pooling and online datasets preprocessing for underwater target detection based on side scan sonar imagery. Remote Sens..

[9] Wen X., Wang J., Cheng C., Zhang F., Pan G. (2024). Underwater side-scan sonar target detection: YOLOv7 model combined with attention mechanism and scaling factor. Remote Sens..

[10] Shi B., Cao T., Ge Q., Lin Y., Wang Z. (2024). Sonar image intelligent processing in seabed pipeline detection: Review and application. Meas. Sci. Technol..

[11] Aubard M., Madureira A., Teixeira L., Pinto J. (2025). Sonar-Based Deep Learning in Underwater Robotics: Overview, Robustness, and Challenges. IEEE J. Ocean. Eng..

[12] Du X., Sun Y., Song Y., Sun H., Yang L. (2023). A comparative study of different CNN models and transfer learning effect for underwater object classification in side-scan sonar images. Remote Sens..

[13] Bai Z., Xu H., Ding Q., Zhang X. (2024). Side-scan sonar image classification with zero-shot and style transfer. IEEE Trans. Instrum. Meas..

[14] Motylinski M., Plater A.J., Higham J.E. (2024). Computer vision methods for side scan sonar imagery. Meas. Sci. Technol..

[15] Sethuraman A.V., Sheppard A., Bagoren O., Pinnow C., Anderson J., Havens T.C., Skinner K.A. (2025). Machine learning for shipwreck segmentation from side scan sonar imagery: Dataset and benchmark. Int. J. Robot. Res..

[16] Tolie H.F., Ren J., Chen R., Zhao H., Elyan E. (2025). Blind sonar image quality assessment via machine learning: Leveraging micro-and macro-scale texture and contour features in the wavelet domain. Eng. Appl. Artif. Intell..

[17] da Silva A.A., Lovisolo L., Ferreira T.N. (2025). Machine Learning Ship Classifiers for Signals from Passive Sonars. Appl. Sci..

[18] Orescanin M., Olson D., Harrington B., Geilhufe M., Hansen R.E., Duvio D., Warakagoda N. (2025). Classification of Imaging Artifacts in Synthetic Aperture Sonar with Bayesian Deep Learning. IEEE J. Ocean. Eng..

[19] Xu F., Huang J., Wu J., Jiang L. (2022). Active mask-box scoring r-cnn for sonar image instance segmentation. Electronics.

[20] Xiao Y., Dai D., Wang H., Li C., Song S. (2025). SDA-Mask R-CNN: An Advanced Seabed Feature Extraction Network for UUV. J. Mar. Sci. Eng..

[21] Li L., Li Y., Wang H., Yue C., Gao P., Wang Y., Feng X. (2024). Side-scan sonar image generation under zero and few samples for underwater target detection. Remote Sens..

[22] Wen X., Zhang F., Cheng C., Hou X., Pan G. (2024). Side-scan sonar underwater target detection: Combining the diffusion model with an improved yolov7 model. IEEE J. Ocean. Eng..

[23] Aubard M., Antal L., Madureira A., Ábrahám E. (2024). Knowledge distillation in YOLOX-ViT for side-scan sonar object detection. arXiv.

[24] Li L., Li Y., Yue C., Xu G., Wang H., Feng X. (2023). Real-time underwater target detection for AUV using side scan sonar images based on deep learning. Appl. Ocean Res..

[25] Wu H., Huang P., Zhang M., Tang W. (2023). CTFNet: CNN-transformer fusion network for remote-sensing image semantic segmentation. IEEE Geosci. Remote Sens. Lett..

[26] Chen M., Zhang Q., Ge X., Xu B., Hu H., Zhu Q., Zhang X. (2023). A full-scale connected CNN–transformer network for remote sensing image change detection. Remote Sens..

[27] Girshick R. (2015). Fast r-cnn. Proceedings of the IEEE International Conference on Computer Vision.

[28] Chai B., Nie X., Zhou Q., Zhou X. (2024). Enhanced cascade R-CNN for multiscale object detection in dense scenes from SAR images. IEEE Sens. J..

[29] Xu X., Zhao M., Shi P., Ren R., He X., Wei X., Yang H. (2022). Crack detection and comparison study based on faster R-CNN and mask R-CNN. Sensors.

[30] Yang F., Huang L., Tan X., Yuan Y. (2024). FasterNet-SSD: A small object detection method based on SSD model. Signal Image Video Process..

[31] Peng H., Li Z., Zhou Z., Shao Y. (2022). Weed detection in paddy field using an improved RetinaNet network. Comput. Electron. Agric..

[32] Zhu M., Hu G., Zhou H., Wang S., Feng Z., Yue S. (2022). A ship detection method via redesigned FCOS in large-scale SAR images. Remote Sens..

[33] AlDahoul N., Karim H.A., De Castro A., Tan M.J.T. (2022). Localization and classification of space objects using EfficientDet detector for space situational awareness. Sci. Rep..

[34] Zhang H., Tian M., Shao G., Cheng J., Liu J. (2022). Target detection of forward-looking sonar image based on improved YOLOv5. IEEE Access.

[35] Ma L., Zhao L., Wang Z., Zhang J., Chen G. (2023). Detection and counting of small target apples under complicated environments by using improved YOLOv7-tiny. Agronomy.

[36] Wang A., Qian W., Li A., Xu Y., Hu J., Xie Y., Zhang L. (2024). NVW-YOLOv8s: An improved YOLOv8s network for real-time detection and segmentation of tomato fruits at different ripeness stages. Comput. Electron. Agric..

[37] Wang Y., Rong Q., Hu C. (2024). Ripe tomato detection algorithm based on improved YOLOv9. Plants.

[38] Wang A., Chen H., Liu L., Chen K., Lin Z., Han J., Ding G. (2024). Yolov10: Real-time end-to-end object detection. Adv. Neural Inf. Process. Syst..

[39] Wang S., Xia C., Lv F., Shi Y. (2025). RT-DETRv3: Real-time end-to-end object detection with hierarchical dense positive supervision. Proceedings of the 2025 IEEE/CVF Winter Conference on Applications of Computer Vision (WACV).

[40] Selvaraju R.R., Cogswell M., Das A., Vedantam R., Parikh D., Batra D. (2017). Grad-cam: Visual explanations from deep networks via gradient-based localization. Proceedings of the IEEE International Conference on Computer Vision.

